# 重组人血管内皮抑制素联合贝伐珠单抗体内抑瘤作用的效果及分析

**DOI:** 10.3779/j.issn.1009-3419.2013.02.01

**Published:** 2013-02-20

**Authors:** 牛 牛, 宝兰 李, 朝阳 刘, 瑛 胡, 雪冰 李, 杰 李, 鹤龄 史, 海清 张

**Affiliations:** 1 101149 北京，北京市结核病胸部肿瘤研究所，首都医科大学附属北京胸科医院综合科 Department of Medical Oncology, Beijing Chest Hospital Affiliated Capital Medical University, Beijing Tuberculosis and Thoracic Tumor Research Institute, Beijing 101149, China; 2 100021 北京，中国医学科学院肿瘤研究所生物检测中心 Tumor Mark Research Center of Cancer Institute & Hospital Chinese Academy of Medical Sciences, Beijing 100021, China; 3 101149 北京，首都医科大学附属北京胸科医院病理科 Department of Pathology, Beijing Chest Hospital Affiliated Capital Medical University, Beijing Tuberculosis and Thoracic Tumor Research Institute, Beijing 101149, China

**Keywords:** 肺肿瘤, 抗血管治疗, 重组人血管内皮抑制素, 贝伐珠单抗, Lung neoplasms, Antiangiogenesis, Endostatin, Bevacizumab

## Abstract

**背景与目的:**

研究重组人血管内皮抑制素和贝伐珠单抗在体内对肺腺癌抑制作用的差别及联合用药的效果。

**方法:**

首先建立A549肺腺癌细胞系的荷瘤Balb/c小鼠动物模型，然后将小鼠随机分为4组，对照组使用普通生理盐水每日瘤周注射。重组人血管内皮抑制素治疗组使用重组人血管内皮抑素（3 mg/kg）每日瘤周注射连续16天贝伐珠单抗治疗组使用贝伐珠单抗（5 mg/kg）每周两次瘤周注射给药。贝伐珠单抗、重组人血管内皮抑制素联合用药组使用贝伐珠单抗（5 mg/kg）每周两次瘤周注射给药+重组人血管内皮抑素（3 mg/kg）每日瘤周注射给药。治疗16天后处死所有实验鼠切取肿瘤标本比较实体瘤大小，采用Western blot的方法检测血管内皮生长因子A和C（vascular endothelial growth factor/VEGF-A, C）在各组表达情况的差异。

**结果:**

重组人血管内皮抑制素和贝伐珠单抗在体内实验中均表现出了抑制肿瘤生长的作用，贝伐珠单抗作用更加明显（52.36% *vs* 38.68%）。联合使用可获得更好的效果（64.15%）。贝伐珠单抗只对VEGF-A有抑制作用（60.8%），重组人血管内皮抑制素对VEGF-A/C都有抑制作用（14.6%, 30.3%）。联合用药组对VEGF-A/C的抑制作用最强（79.4%, 44.2%）。

**结论:**

重组人血管内皮抑制素和贝伐珠单抗都在裸鼠动物模型试验中表现出了明显的抑瘤效果，联合使用抑制肿瘤效果更加明显。恩度对肿瘤组织内VEGF-A/C都表现出了抑制作用贝伐珠单抗对VEGF-A表现出了较明显的抑制作用联合用药组对VEGF-A/C抑制作用更加明显。

## Abstract (en)

**Background and objective:**

The aim of this study is to investigate difference of antiangiogenesis ablility and interaction about bevacizumab and endostatin *in vivo* in lung cancer animal model.

**Methods:**

First, we construct a Balb/c mice model with A549 lung adenocarcinoma cell. Then, we divide the mice into four groups randomly. Every group has six mice. Control group: mice injected with normal saline every day aroud the tumor. Endostatin group: mice injected with endostatin (Recombinant endostatin) injection (3 mg/kg) every day Peritumoral. Bevacizumab group: mice injected with bevacizumab twice a week (biw/5 mg/kg) Peritumoral. Combing group: mice were injected with endostatin and bevacizumab (dose just like single drug group). After 16 days, we executed mice and got the tumor tissue for next analysis.

**Results:**

Based on this experiment, we found bevacizumab and endostatin expressed the ability for inhibiting tumor growth *in vivo*. Bevacizumab was more powerful (52.36% *vs* 38.68%). Combining bevacizumab with endostatin could get better results (64.15%) than single drug did. Bevacizumab played the role by inhibiting VEGF-A expression (60.8%). Endostatin took effect by inhibiting VEGF-A/C (14.6%, 30.3%). Combining group present better antitumor efficacy than any single drug group did. (79.7%, 44.2%).

**Conclusion:**

Both bevacizumab and endostatin present the antiangiogenesis ability *in vivo* of lung cancer animal model. In our test, bevacizumab show better ability in inhibiting tumor growth than endostatin does. Additional, combing bevacizumab with endostatin reveal better potential to inhibit tumor growth than single drug does.

自20世纪70年代Folkman教授^[1]^提出肿瘤生长和转移依赖于肿瘤内部新生血管的观点后，很多研究一直致力于通过阻断肿瘤新生血管形成来达到治疗肿瘤的目的。抗血管治疗近年来随着靶向治疗在肿瘤治疗领域的方兴未艾，越来越成为临床治疗肿瘤的重要受手段之一。肺癌则是世界上对人类健康威胁最大的一种常见肿瘤，目前世界上每年大约新发肺癌病例约160万人^[2]^，而肺癌的治疗效果目前仍然很不理想，据报道，美国每年因肺癌死亡人口超过16万^[3]^。近年来随着靶向药物和抗血管治疗药物进入临床，延长了肺癌特别是中晚期肺癌患者的生存期。目前在国内外最常见的肿瘤抗血管治疗药物就是贝伐珠单抗和重组人血管内皮抑制素（及其改良制剂）。两种药物作用机制各异，虽然有很多体内外实验都证明两种药物有明确的抗肿瘤作用，但是目前尚缺乏在同一个实验模型和条件下，对两种药物实际作用效果的分析的实验。我们也注意到在国内外的多中心临床研究中抗血管治疗只使用一种药物，虽然研究结果证明抗血管治疗可以延长患者的生存期，但是作用还很有限^[4]^。本实验第一次在同一试验模型下比较重组人血管内皮抑制素和贝伐珠单抗在体内实际的抗肿瘤效果。同时第一次联合使用两种抗血管药物，分析有无联合用药的可能性，探索将来肿瘤临床抗血管治疗的新方法和新思路。

## 1 实验方法

### 1.1 主要药物及实验材料

重组人血管内皮抑制素注射液（恩度，Endostar），购自山东先声麦得津生物制药有限公司。贝伐珠单抗注射液（安维汀，Avastin），购自上海罗氏制药有限公司。RIPA蛋白抽提试剂盒及BCA蛋白定量试剂盒购自北京赛驰生物科技公司。VEGF兔抗人单克隆抗体（IgG VEGF-A、IgG VEGF-C）抗体购自北京新新通生物科技公司。凝胶分析系统采用复日Smart View生物电泳图像分析系统。实验动物：Balb/c裸鼠（16 g-18 g，SPF级）中国药品生物制品检定所实验动物中心提供（实验动物质量合格证号SCXK（京）2009-0017）。人肺腺癌A549细胞株，由D.J. Giard等建株，中国医学科学院肿瘤研究所提供。

### 1.2 实验方法

#### 1.2.1 体外细胞培养

A549细胞常规培养于含10%胎牛血清、100 U/mL青霉素、100 U/mL链霉素的DMEM培养基中，置37 ℃、饱和湿度、5%CO_2_的培养箱中培养，2天传代一次，取对数生长期细胞用于实验。

#### 1.2.2 体内动物实验

取经体外细胞培养已在裸鼠皮下传代生长良好的移植性肺腺癌肿瘤结节，无菌操作制成瘤细胞小块，用穿刺针接种于Balb/c裸鼠腋窝皮下，1块/鼠（约2 mm^3^）。将动物随机分为4组，每组6只，称体重标号。4组分别为模型对照组（control group, CG）、重组人血管内皮抑制素（3 mg/kg）组（Endostatin group, EG）、贝伐珠单抗（5 mg/kg）（Bevacizumab group, BG）组、重组人血管内皮抑制素（3 mg/kg）联合贝伐珠单抗（5 mg/kg）（combining group, COG）组。各组在接种肿瘤后第15日开始皮下瘤周给药，重组人血管内皮抑制素每天1次，连续16天，共16次。贝伐珠单抗每周2次（周一、周二给药），共6次。模型对照组使用0.9%无菌氯化钠液（5 mL/kg）每日瘤周注射，共16次。重组人血管内皮抑制素（3 mg/kg）、贝伐珠单抗（5 mg/kg）联合用组给药方式同单独给药组。裸鼠饲养室拥有IVC-Ⅱ型（智能型）独立送风隔离笼具，室温20 ℃-22 ℃，相对湿度40%-60%（实验动物使用许可证号SYXK(京)2008-0025）。裸鼠食用灭菌裸鼠饲料，SPF级，中国医学科学院实验动物研究所产品（许可证号SCXK(京)2009-0008），执行标准GB14924.3-2001。实验开始后每4天用卡尺测一次皮下肿瘤体积，每次给药结束后观察荷瘤鼠表现，于肿瘤接种后第31天处死动物，完整剖取瘤结并称瘤重和体重，计算相对肿瘤增殖率。

#### 1.2.3 Western blot实验

将肿瘤标本用含有蛋白酶抑制剂的RIPA蛋白抽提试剂盒裂解，而后于冰上孵育20 min，4 ℃离心（13, 000 rpm, 20 min）。离心完成后取上清经BCA蛋白质浓度测定试剂盒进行蛋白质浓度测定。10%SDS聚丙烯酰胺凝胶电泳分离蛋白，然后转至固相载体（NC膜）上。10%牛奶封闭非特异抗原，加入一抗（兔抗人VEGF-A/C单克隆抗体）4 ℃反应过夜，常温洗膜后加入二抗，室温反应1 h。用Pierce公司的Supersignal West Dura Extended Duration Substrate ECL化学发光系统进行检测，将A液与B液等体积混合，配成发光剂，滴在保鲜膜上，将NC膜倒扣于其上，5 min后用另一保鲜膜将NC膜包裹，X线片压片，曝光20 s-5 min，显影2 min，定影2 min。Western blot结果扫描储存。用复日Smart View生物电泳图像分析系统分析结果。

#### 1.2.4 药效判定标准：

$
 肿瘤抑制率 = (1 - \frac{{给药组平均瘤重\left( {\rm{T}} \right)}}{{对照组平均瘤重\left( {\rm{C}} \right)}}) \times 100\% \\
肿瘤体积\left( {\rm{V}} \right) = 肿瘤长径\left( {\rm{L}} \right) \times 肿瘤短径{\left( {\rm{S}} \right)^2}/2
			$

按以下公式计算相对肿瘤体积（RTV）和相对肿瘤增殖率T/C%：RTV=Vt/V0

Vt：测量肿瘤得到的瘤体积

V0：初始瘤体积（给药前）

T/C%=给药组的RTV平均值/对照组的RTV平均值×100%

$
			{\rm{Q = }}\frac{{{{\rm{E}}_{{\rm{AB}}}}\left( {实测值} \right)}}{{{{\rm{E}}_{\rm{A}}} + {{\rm{E}}_{\rm{B}}} - {{\rm{E}}_{\rm{A}}} \cdot {{\rm{E}}_{\rm{B}}}\left( {期望值} \right)}}
			$

公式中E_A_为A药抑瘤率，E_B_为B药抑瘤率，E_AB_为两药合用抑瘤率，q=0.85-1.15为两药合用作用相加，q＞1.15为两药合用作用增强，q＜0.85为两药合用作用拮抗^[5]^。

Western blot结果分析：采用凝胶图像分析系统，对电泳条带进行密度扫描后进行灰度分析（Smart View生物电泳图像分析系统）。

#### 1.2.5 统计方法

统计数据处理均采用SPSS 13.0软件系统处理，动物实验结果所有数据以Mean±SD表示，组间比较采用*t*检验，*P*＜0.05为差异有统计学意义。

## 2 结果

### 2.1 动物实验结果

实验共计31天，实验结束时重组人血管内皮抑制素（3 mg/kg）联合贝伐珠单抗（5 mg/kg）组抑瘤率为64.15%，与对照组比较差异有统计学意义（*P*＜0.01），重组人血管内皮抑制素单药组（3 mg/kg）抑瘤率为38.68%，贝伐珠单抗单药组（5 mg/kg）抑瘤率为52.36%。重组人血管内皮抑制素、贝伐珠单抗联合用药Q值为0.906，提示两药联合使用作用相加。实验结束，各治疗组动物与对照组比较，体重无明显差异，动物活动正常未表现毒性反应。表 1显示各组实验动物基本情况和实验数据。给药期间肿瘤生长曲线见图 1。实验结束时各组瘤重图见图 2。实验结束时的肿瘤组织瘤结照片见图 3。

**1:** 动物实验基本情况 Basic information about the experiment

Group	Dose (mg/kg)	Number of mice		Frequency position	MW (Mean±SD, g)		TW (Mean±SD, g)	T/C (%)	TIR	*P* ^#^
		B	E		B	E				
CG	N.S.	6	6	Perit×16 qd	5.2±1.1	25.7±1.1	0.35±0.06			
EG	3	6	6	Perit×16 qd	25.3±1.2	25.8±1.1	0.22±0.10	64.83	38.68	< 0.01
BG	5	6	6	Perit×6 Biw	25.4±1.4	26.0±0.8	0.17±0.07	50.74	52.36	< 0.01
COG	3	6	6	Perit×16 qd	24.4±1.2	25.6±0.4	0.13±0.07	41.30	64.15	< 0.01
	5			Perit×6 Biw						
NS: sterile normal saline; Biw: bis in week; #compared with control group; Perit: Peritumoral injection; CG: control group; EG: endostatin group; BG: bevacizumab group; COG: combining group; MW: mice weight; TW: tumor weight; B: before treatment; E: end of experiments; TIR: tumor inhibition rate; T/C (%) proliferation rate.										

**1:**
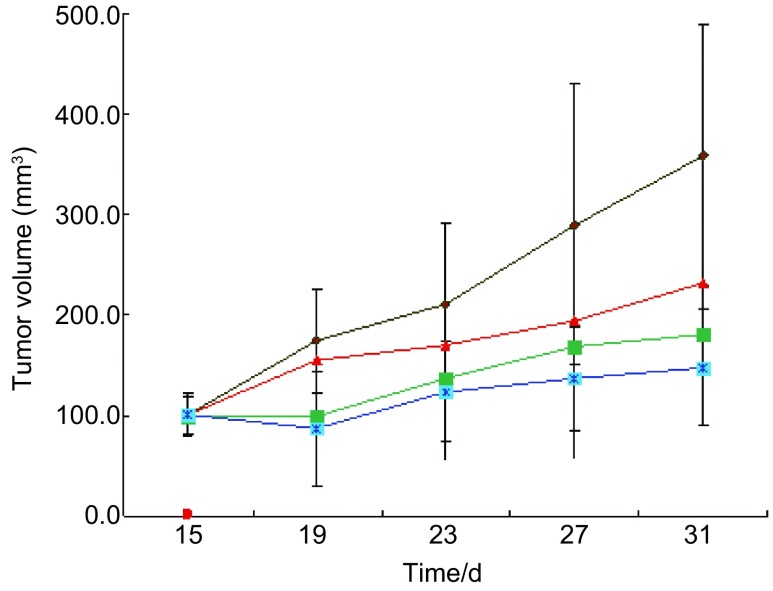
各组间肿瘤体积生长情况分析(棕色曲线：对照组平均种瘤体积（347.27±3.51）mm^3^；红色曲线：重组人血管内皮抑制素治疗（198.29±2.13）mm^3^；绿色曲线：贝伐珠单抗治疗组（143.57±2.82）mm^3^；蓝色曲线：联合用药组（117.63±1.93）mm^3^。各组较对照组相比均有统计学差异，*P*＜0.01)。 Tumor survival time after administration of endostatin or combined with bevacizumab on nude mice bearing A549. Brown line: control group: (347.27±3.51) mm^3^; Red line: endostatin group: (198.29±2.13) mm^3^; Green line: bevacizumab group: (143.57±2.82) mm^3^; blue line: combining group: (117.63±1.93) mm^3^. Compared with control group, *P* < 0.01).

**2:**
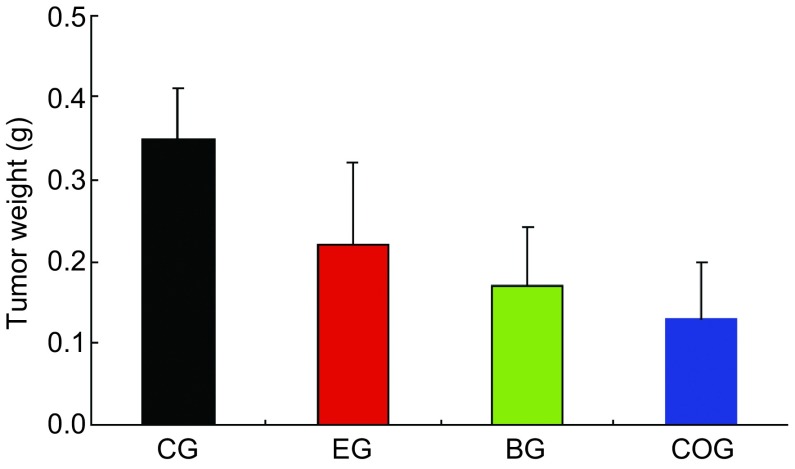
各组间抑瘤效果分析。黑色柱：对照组肿瘤平均重量（0.35±0.06）g；红色柱：重组人血管内皮抑制素治疗组肿瘤平均重量（0.22±0.10）g，抑瘤率：38.68%；绿色柱：贝伐珠单抗治疗组肿瘤平均重量（0.17±0.07）g，抑瘤率：52.36%；蓝色柱：联合治疗组肿瘤平均重量（0.13±0.07）g，抑瘤率：64.15%。各组较对照组相比均有显著差异，*P*＜0.01）。 Inhibitive effect of endostatin combined with bevacizumab on nude mice bearing A549. Black column: control group, TW: (0.35±0.06) g; Red column: endostatin column, TW: (0.22±0.10) g, TIR: 38.68%; Green column: bevacizumab column, TW: (0.17±0.07) g, TIR: 52.36%; Blue column: combining group, TW: (0.13±0.07) g, TIR: 64.15%. Compared with control group, *P* < 0.01).

**3:**
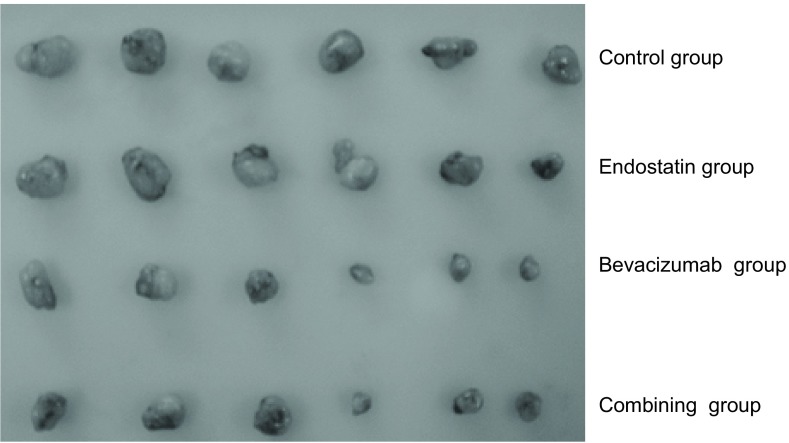
瘤体切除后照片 Photograph of Endostatin combined with bevacizumab on nude mice bearing transplanted tumor A549

### 2.2 Western blot实验结果

对Western blot电泳结果分析：贝伐珠单抗组VEGF-A表达较对照组明显减少，抑制率为60.8%。重组人血管内皮抑制素组VEGF-A（图 4）、VEGF-C（图 5）表达较对照组亦有减少，抑制率分别为14.6%、30.3%。重组人血管内皮抑制素联合贝伐珠单抗组VEGF-A、VEGF-C表达较对照组抑制最为显著，抑制率为79.7%、44.2%。β-Actin表达在各组中无明显差异。

**4:**
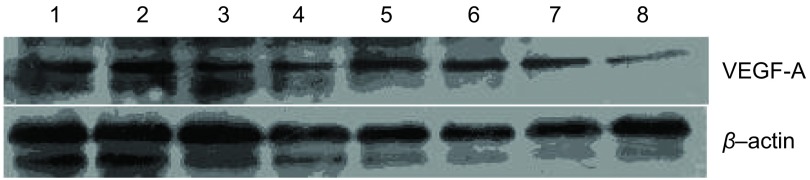
Western blot检测各组中VEGF-A蛋白的表达。泳道1、2：对照组；泳道3、4：贝伐珠单抗组，抑制率60.8%；泳道5、6：重组人血管内皮抑制素治疗组，抑制率14.6%；泳道7、8：联合治疗组，抑制率79.7%。实验数据均采用复日smart view生物电泳图像分析系统处理。 Western blot analysis for VEGF-A protein expression. Line 1, 2: control group; Line 3, 4: bevacizumab group/inhibition rate (IR): 60.8%; Line 5, 6: endostatin group/IR: 14.6%; Line 7, 8: combining group/IR: 79.7%. All date was analyzed by smart view system and compared with control group.

**5:**
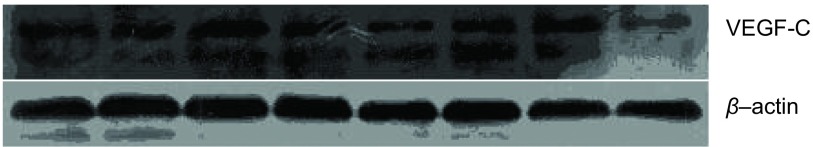
Western blot检测各组中VEGF-C蛋白的表达。泳道1、2：对照组；泳道3、4：贝伐珠单抗组，较对照组无明显改变；泳道5、6：重组人血管内皮抑制素治疗组抑制率30.3%；泳道7、8：联合治疗组，抑制率44.2%。实验数据均采用复日Smart View生物电泳图像分析系统处理。 Western blot analysis for VEGF-C protein expression. Line 1, 2: control group; Line 3, 4: bevacizumab group, no difference with control group; Line 5, 6: endostatin group/IR: 30.3%; Line 7, 8: combining group/IR: 44.2%. All date was analyzed by Smart View system and compared with control group.

## 3 讨论

### 3.1 重组人血管内皮抑制素和贝伐珠单抗的相互作用

通过实验我们发现重组人血管内皮抑制素和贝伐珠单抗在体内实验均表现出了一定的抑制肿瘤生长的能力，贝伐珠单抗抑制肿瘤生长的能力更强（52.36% *vs* 38.68%）。联合使用两种药物则效果更为明显（64.15%）。贝伐珠单抗是一个作用机制明确的单克隆抗体药物。其为一个人源化的VEGF单克隆IgG抗体，通过与循环VEGF-A结合，阻止其与内皮细胞表面相应的VEGF受体结合达到抗肿瘤新生血管之作用^[6, 7]^。而重组人血管内皮抑制素自1997年O’ Reilly等^[8]^从小鼠血管内皮细胞瘤的培养上清液中分离发现以后，虽然在众多体内外实验中均发现有明显的抑制肿瘤新生血管形成的作用，但具体作用机制至今仍然未明确。Tan等^[9]^研究发现重组人血管内皮抑制素处于肿瘤新生血管调节网络中的关键部位，能够下调多种促血管形成因子的表达如血管内皮生长因子、成纤维细胞生长因子、表皮生长因子受体等，同时能上调多种抗肿瘤血管形成因子的表达，如血小板反应蛋白1、酪氨酸蛋白激酶B3抗体等。Dhanabal等^[10, 11]^研究发现重组人血管内皮抑制素可能增加血管内皮细胞的凋亡，改变血管内皮细胞的细胞周期。Marko等^[12]^发现固定的重组人血管内皮抑制素促进内皮细胞存活，而可溶性的重组人血管内皮抑制素则促进其凋亡。Nicholas等^[13]^发现重组人血管内皮抑制素可破坏肌球蛋白微丝结构的完整性，导致内皮细胞运动能力下降。Kim等^[14]^研究发现重组人血管内皮抑制素的部分抗肿瘤活性是通过调节基质金属蛋白酶实现的，虽然相关研究很多，但目前尚难就重组人血管内皮抑制素抑制肿瘤新生血管形成的确切机制达成共识。本实验发现重组人血管内皮抑制素和贝伐珠单抗联合使用，药效上有相加作用（Q: 0.969），最近一些研究也表明重组人血管内皮抑制素的部分抗血管生成作用也是通过影响VEGF表达来实现的。Huang等^[15]^发现血管内皮生长因子可促进细胞核内的核仁素迁徙到细胞表面，而用抗体封闭等技术，降低细胞表面表达的核仁素，可以明显降低内皮细胞的迁徙能力，防止新生脉管的形成。Song等^[16]^和Zhou等^[17]^都通过研究也发现核仁素也是重组人血管内皮抑制素发挥抗肿瘤血管和淋巴管生成的重要受体。这些研究表明两种药物在作用机制上的确存在着交集。在本实验中两者表现出作用相加效果，但其具体机制还需要更多的研究才能明确。

### 3.2 对淋巴管和血管的抑制作用

通过Western blot实验结果我们可以看出在体内贝伐珠单抗对VEGF-A的抑制作用明显强于重组人血管内皮抑制素（60.8% *vs* 14.6%），而重组人血管内皮抑制素对VEGF-C则有一定的抑制作用（30.3%）。贝伐珠单抗相对于对照组没有表现出对VEGF-C的抑制作用。这也与贝伐珠单抗是个单一的VEGF-A的单克隆抗体对VEGF-C没有作用有关。众所周知VEGF-C于淋巴管的形成关系密切，而重组人血管内皮抑制素对淋巴管的抑制作用，相关研究也有类似报道^[18]^。联合用药组的实验结果发现无论是对于VEGF-A还是VEGF-C均表现出了较强的抑制作用（79.4% *vs* 44.2%），这也与物实验中各组移植瘤抑瘤率的差异相吻合。我们发现虽然贝伐珠单抗本身对VEGF-C没有直接作用，但是联合使用后，联合用药组VEGF-C的抑制效果却较单独使用重组人血管内皮抑制素更好，与之类似，Véronique Mathieu^[19]^发现在胶质母细胞瘤动物模型中，联合使用贝伐珠单抗和替莫唑胺能够增强后者的抗肿瘤血管生成的作用。但必须强调的是：本实验也仅仅发是现了这一现象，并不能由此即做出贝伐珠单抗能增强重组人血管内皮抑制素抑制淋巴管形成作用的推断。由于肿瘤的脉管调节机制是一个巨大而复杂的网络，目前人类还很难全面了解，所以具体的作用机制还需要更多的研究才能明确。

## 4 结论

通过本实验我们发现了两种抗血管治疗药物在动物体内抗肿瘤作用的差异，同时也发现两者联合使用有相加作用。肿瘤的血管和淋巴管生成是一个复杂的调控网络，本实验的重要性在于第一次在同一个试验模型下对两种常用的抗肿瘤血管生成的药物疗效进行了分析，第一次发现了两种药物联合使用后效果较单独用药更显著，存在相加作用。而动物实验中也并未发现有实验动物因为联合用药出现严重致死的毒副反应。这为以后进一步通过这些现象了解肿瘤脉管形成的复杂调节网络提供了一个新的切入点，也为临床进一步改善抗血管治疗的效果提供了新的思路。
